# Restoring electronic coherence/decoherence for a trajectory-based nonadiabatic molecular dynamics

**DOI:** 10.1038/srep24198

**Published:** 2016-04-11

**Authors:** Chaoyuan Zhu

**Affiliations:** 1 Institute of Molecular Science, Department of Applied Chemistry, and Center for Interdisciplinary Molecular Science, National Chiao-Tung University, Hsinchu 300, Taiwan; 2 State Key Laboratory of Molecular Reaction Dynamics, Dalian Institute of Chemical Physics, Chinese Academy of Science, Zhongshan Road 457, Dalian 116023, P. R. China

## Abstract

By utilizing the time-independent semiclassical phase integral, we obtained modified coupled time-dependent Schrödinger equations that restore coherences and induce decoherences within original simple trajectory-based nonadiabatic molecular dynamic algorithms. Nonadiabatic transition probabilities simulated from both Tully’s fewest switches and semiclassical Ehrenfest algorithms follow exact quantum electronic oscillations and amplitudes for three out of the four well-known model systems. Within the present theory, nonadiabatic transitions estimated from statistical ensemble of trajectories accurately follow those of the modified electronic wave functions. The present theory can be immediately applied to the molecular dynamic simulations of photochemical and photophysical processes involving electronic excited states.

A mixed quantum-classical dynamics starts from solving electronic adiabatic potential energy surfaces *U*_j_(**R**) and nonadiabatic coupling vectors **d**_ij_(**R**) by applying various ab initio quantum chemistry methods. Then, the nuclear motion is represented by classical trajectories computed by numerical integration of Newton’s equations. The nonadiabatic transitions along the classical trajectories are described by the coupled time-dependent Schrödinger equations in terms of the density matrix representation^1^,


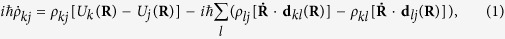


where **R** is an *N*-dimensional vector of nuclear coordinates and an overdot denotes a time derivative (*N*-dimensional velocity) with the sum over all adiabatic electronic states. Tully’s fewest switches^1^ and the semiclassical Ehrenfest^2^ algorithms, which are the two representative methods, provide a powerful tool to perform nonadiabatic molecular dynamics simulations with simple trajectory-based approaches. These trajectory-based nonadiabatic molecular dynamics methods with various modified versions have been successfully applied to photochemical and photophysical related molecular spectra and reaction dynamics for large systems^3^,^4^,^5^,^6^,^7^,^8^,^9^,^10^,^11^,^12^,^13^,^14^,^15^,^16^,^17^. Tully’s fewest switches method propagates nonadiabatic trajectory on each adiabatic electronic state with trajectory surface hopping from one adiabatic potential energy surface to another. The semiclassical Ehrenfest method propagates nonadiabatic trajectory on averaged adiabatic electronic states in which a single mean-field potential energy surface governs nuclear motion. Both methods suffer an overcoherence problem in electronic wavefunction (or density matrix *ρ*_*kj*_ in eq. (1)) when nonadiabatic trajectory passes through non-zero region of nonadiabatic coupling vector. In order to reproduce exact coherent motion of quantum wavefunction propagating on multiple adiabatic electronic states, the semiclassical density matrix described by eq. (1) should decohere. Numerous algorithmic coherence/decoherence schemes in the literature have been designed for nonadiabatic trajectory coupled with electronic motion^18^,^19^,^20^,^21^,^22^,^23^,^24^,^25^,^26^,^27^,^28^,^29^,^30^,^31^,^32^. All of these approaches can improve decoherence/coherence effects of the electronic wavefunction to some extent but at either high computational cost or through the use of complicated algorithms.

## Results

### Theory

In order to preserve simplicity of trajectory-based algorithms for large-scale nonadiabatic molecular dynamic simulations, we propose both simple and accurate scheme to restore electronic coherence/decoherence in a unified way for both Tully’s fewest switches and the semiclassical Ehrenfest methods. Let us exam solution of eq. (1) in the regions where nonadiabatic coupling vector is equal to zero, we have


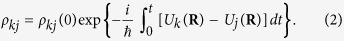


Let us compare the phase integral in eq. (2) with the conventional time-independent Jeffreys-Wentzel-Kramers-Brillouin^33^ (JWKB) semiclassical phase integral


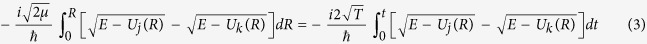


where *μ* is reduced mass, *E* is total energy and *T* = (*E − U*) is kinetic energy. From trajectory-based approach, *R* in eq. (3) can be considered as a classical trajectory propagating along a one-dimensional curved space. Alternatively, we can obtain the same relation as


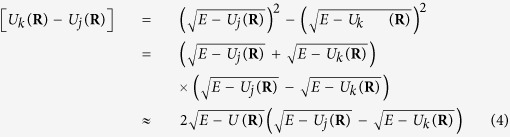


in which *U*(**R**) is an effective potential energy surface for nonadiabatic trajectory. For Tully’s fewest switches approach, *U*(**R**) is a single adiabatic potential energy surface *U*_*j*_(**R**) or *U*_*k*_(**R**) on which trajectory is propagating, and for semiclassical Ehrenfest approach, *U*(**R**) is defined as an average potential energy surface


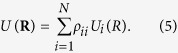


Finally, we obtain modified coupled time-dependent Schrödinger equations,


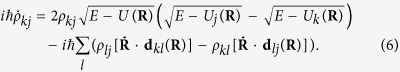


Equation (4) shows that time-dependent and time-independent approaches have the same limit in the high-energy regime. We can expect that both original and modified coupled Schrödinger equations should agree each other for the high-energy regime, namely *E − U *≫ |*U*_k_(**R**) − U_j_ (**R**)|).

Using a similar approach to the semicalssical Ehrenfest method, the symmetrical windowing quasi-classical approach^27^ (that is as simple as the present theory) restores coherence/decoherence pretty well with windowing parameter γ = 0.366. However, numerical tests in Supplementary Note 1 show that the present modified Ehrenfest approach is slightly better than the symmetrical windowing quasi-classical approach. Using a similar approach to the Tully’s fewest switches trajectory surface hopping method, the Gaussian wavepackets phase correlation method^28^ (that is more complicated than the present theory) restores coherence/decoherence in a similar way as the present one, and actually two are the same in one-dimensional case. However, the two are different in multidimensional case, their diagonal element in Hamiltonian does not approach limit *U*_k_(**R**)−U_j_ (**R**) in the high-energy regime, and their decoherent term (**p**_**1**_ **· p**_**2**_**/**m) requires calculation of classical momentum on two adiabatic potential energy surfaces simultaneously. Numerical tests in Supplementary Note 3 show that the present modified the Tully’s fewest switches approach can nicely reproduce quantum oscillation for the certain two-dimensional model system.

We select four well-known model problems in the following to perform both semiclassical Ehrenfest and Tully’s fewest switches calculations from original eq. (1) and modified eq. (6). Model 1 describes the electronic transitions in the non-crossing case of adiabatic potential energy surfaces initially developed by Rosen and Zener^34^. Model 2 and Model 3 describe the electronic transitions in the avoided-crossing case of adiabatic potential energy surfaces initially developed by Landau^35^, Zener^36^ and Stückelberg^37^. Model 4 describes the electronic transitions in the crossing case with the peculiar degeneracy of adiabatic potential energy surfaces initially developed by Renner^37^,^38^. All these pioneer studies focused on developing analytical formula for nonadiabatic transition probability from various mathematical methods nicely documented by Child^39^. The present study focuses on simulating nonadiabatic transition probability for all cases numerically solving original eq. (1) and modified eq. (6).

We compute the overall nonadiabatic transition probability defined as the probability of starting on the lower adiabatic potential energy surface at *x* = *−*∞ and finishing on the upper adiabatic potential energy surface at *x* = +∞. Accurate quantum mechanical calculations for the four one-dimensional two-state models are performed using the conventional time independent close-coupling method. Semiclassical calculations are performed by using the fourth-order Runge–Kutta method for numerically integrating the trajectories as well as coupled time-dependent Schrödinger equations. For a given total energy, the Tully’s fewest switches method unitizes 10,000 sampling trajectories while the Ehrenfest semiclassical method needs only one sampling trajectory. The reduced mass is chosen to be 2000 au for all four model systems.

### Dual Rosen-Zener-Demkov non-crossings

**Figure 1:**
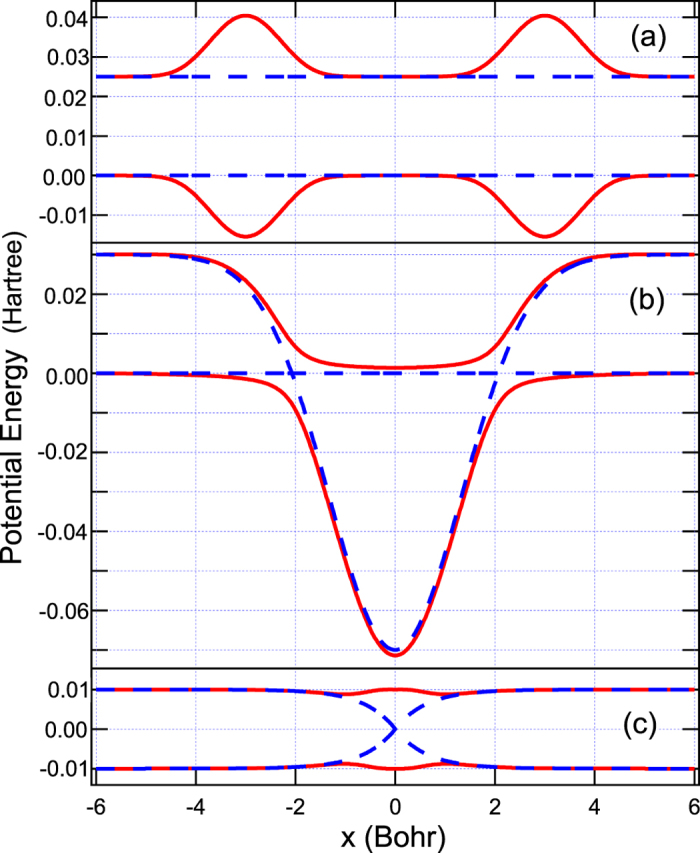
Potential energy curves for (**a**) Model1, (**b**) Model 2 and (**c**) Model 3. Dashed (solid) lines represent diabatic (adiabatic) potential energy curves.

**Figure 2:**
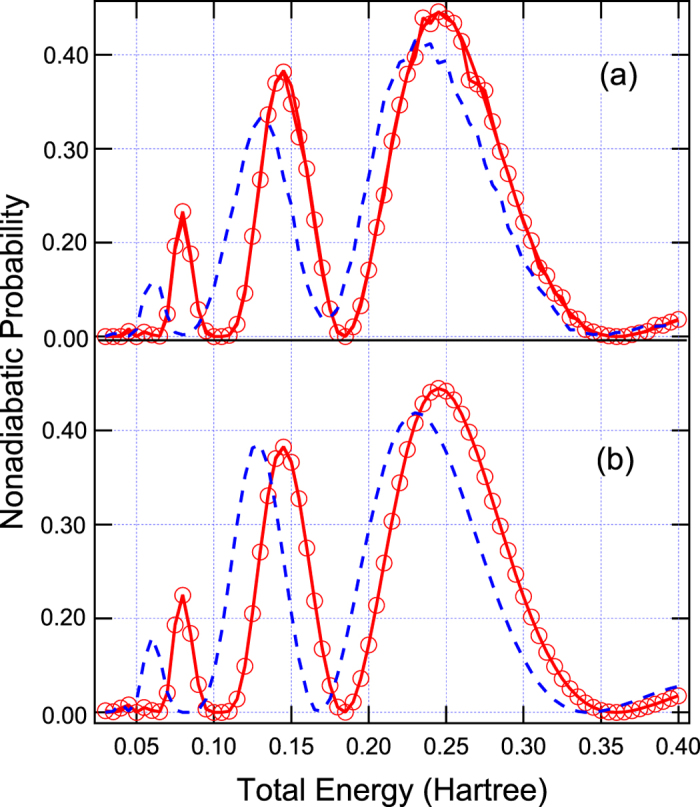
Overall nonadiabatic transition probabilities for Model 1 are calculated from (**a**) Tully’s fewest switches and (**b**) semiclassical Ehrenfest methods. Dashed lines represent original semiclassical results (see eq. (1)), open circles represent the present modified semiclassical results (see eq. (6)), and solid lines represent exact quantum results.

Model 1 is defined by two parallel diabatic potentials with diabatic coupling expressed in terms of two Gaussian functions:


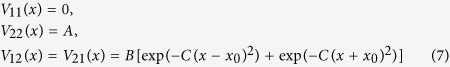


with the parameters chosen to be *A* = *B* = 0.025 Hartree, *x*_0_ = 3.0 Bohr, and *C* = 0.7 Bohr^−2^. This model can induce electronic coherence from overlapping of two semiclassical wavepackets in between two non-crossing peaks at *x* = ±3.0 Bohr as shown in Fig. 1(a). The results simulated by Tully’s fewest switches (see Fig. 2(a)) and semiclassical Ehrenfest (see Fig. 2(b)) within the modified semiclassical eq. (6) follow exact quantum oscillations and amplitudes of the overall nonadiabatic transition probabilities very well. The results simulated from the original semiclassical eq. (1) cannot reproduce exact quantum results, and besides Tully’s fewest switches and semiclassical Ehrenfest methods do not agree each other for oscillations and amplitudes of the overall nonadiabatic transition probabilities.

### Dual Landau-Zener-Stückelberg avoided-crossings

**Figure 3:**
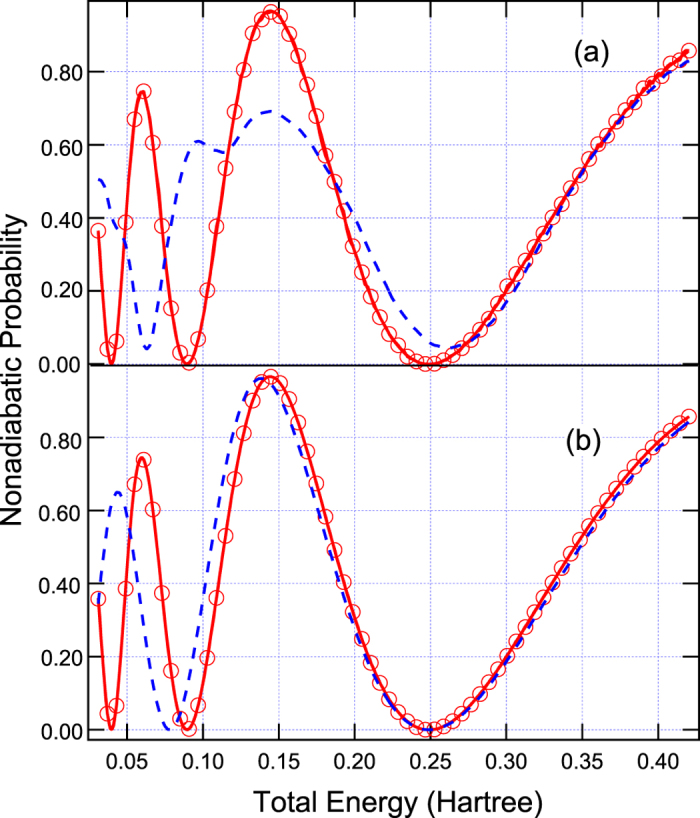
The same as Fig. 2 except for Model 2. (**a**) Tully’s fewest switches and (**b**) semiclassical Ehrenfest methods.

Model 2 is defined by two diabatic potentials having dual crossings given in the diabatic representation:


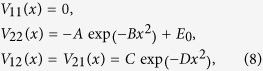


with the parameters chosen to be *A* = 0.1 Hartree, *B* = 0.28 Bohr^−2^, *E*_0_ = 0.03 Hartree, *C* = 0.01 Hartree, and *D* = 0.06 Bohr^−2^. This model can induce different electronic coherence from overlapping of two semiclassical wavepackets in between two avoided-crossings at *x* = ±2.07 Bohr as shown in Fig. 1(b). The results simulated by Tully’s fewest switches (see Fig. 3(a)) and semiclassical Ehrenfest (see Fig. 3(b)) within the modified semiclassical eq. (6) follow exact quantum oscillations and amplitudes of the overall nonadiabatic transition probabilities very well. The results simulated from the original semiclassical eq. (1) cannot reproduce exact quantum results, and besides Tully’s fewest switches and semiclassical Ehrenfest methods show very different oscillations and amplitudes of the overall nonadiabatic transition probabilities. This model system has been well studied in the literatures for the subject of decoherence problem. We show in Supplementary Note 2 that if we shrink *E*_0_ = 0.015 in eq. (8) by well satisfying (*E* − *U*) ≫ *U*_2_(**R**) − U_1_ (**R**) = *E*_0_, the overall nonadiabatic transition probabilities calculated from both original eq. (1) and the present eq. (6) all agree with exact quantum results in the high-energy regime. However, in low energy region, the present theory (see Supplementary Figure S3) works very well but the original one (see Supplementary Figure S4) fails. Two statistical averaged populations based on number of trajectories and electronic wavefunction agree each other for Tully’s fewest switches approach with either equations (1) or (6).

### Simple avoided crossing

**Figure 4:**
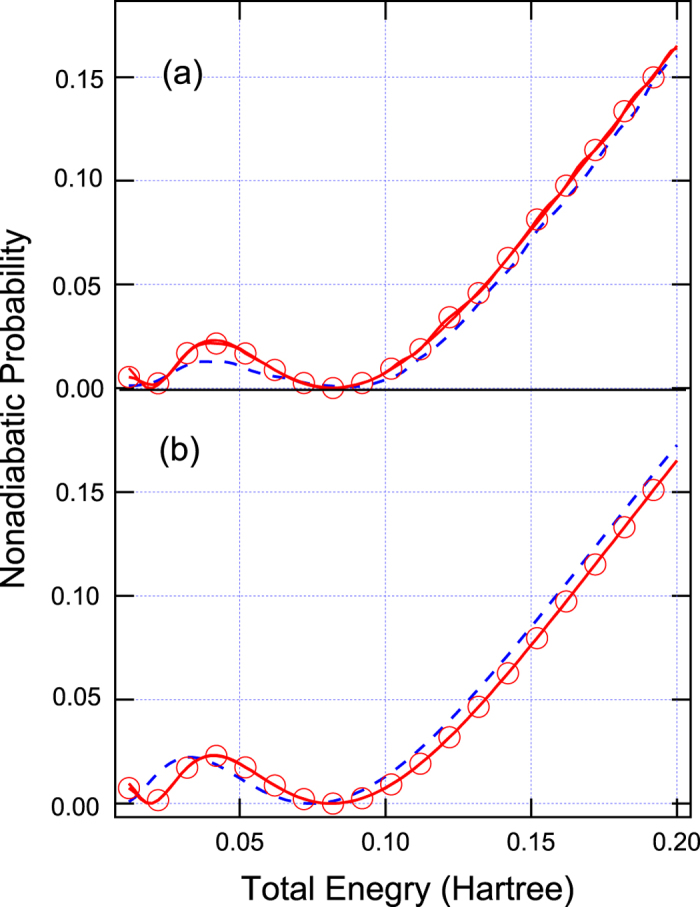
The same as Fig. 2 except for Model 3. (**a**) Tully’s fewest switches and (**b**) semiclassical Ehrenfest methods.

Model 3 is defined by two diabatic potentials having one simple crossing given in the diabatic representation:


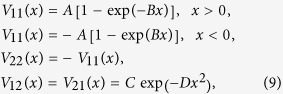


with *A* = C = 0.01 Hartree, *B* = 1.6 Bohr^−1^, and *D* = 1.0 Bohr^−2^. One simple diabatic crossing is appeared at *x* = 0 as shown in Fig. 1(c). However, the strong Gaussian-type diabatic coupling can produce two vague avoided crossings around *x* = ±1.0 Bohr, and thus it can induce small electronic coherence at low energies as shown in Fig. 4 The results simulated by Tully’s fewest switches (see Fig. 4(a)) and semiclassical Ehrenfest (see Fig. 4(b)) within the modified semiclassical eq. (6) can well reproduce this small oscillation and amplitudes of nonadiabatic transition probabilities. The results simulated from the original semiclassical eq. (1) cannot well reproduce this small oscillation, although Tully’s fewest switches and semiclassical Ehrenfest methods almost agree with the exact quantum amplitudes of the overall nonadiabatic transition probabilities for high energies, but two are not exactly the same.

### Renner-Teller crossing

**Figure 5:**
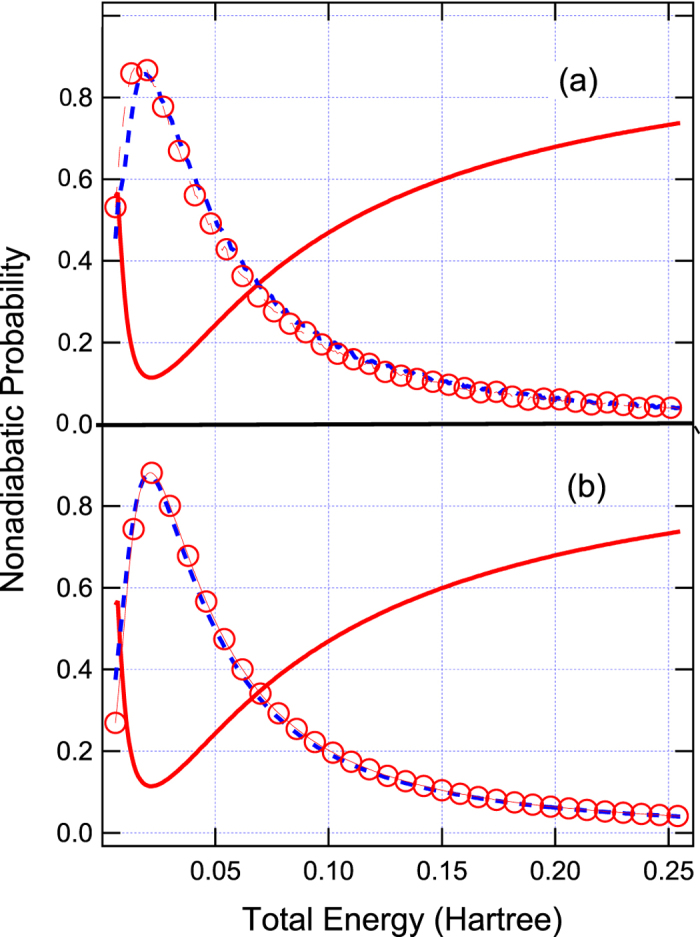
The same as Fig. 2 except for Model 4. (**a**) Tully’s fewest switches and (**b**) semiclassical Ehrenfest methods.

Model 4 is defined by two diabatic potentials having same as *V*_11_(*x*) and *V*_22_(*x*) in eq. (9). However, diabatic coupling is changed as


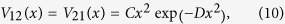


in which diabatic coupling approaches zero as *x*^2^ at diabatic crossing point *x* → 0. This is typical Renner-Teller coupling^40^. Potential parameters are chosen to be *A* = 0.005, C = 0.01 Hartree, *B* = 1.6 Bohr^−1^, and *D* = 1.0 Bohr^−2^. Due to significant degeneracy of two adiabatic potential energy surfaces at crossing zone, the both original and modified methods cannot follow exact calculation of the overall nonadiabatic transition probability as shown in Fig. 5. This means that the restoring term in eq. (6) is still not good enough for describing electronic transitions in significant degenerate case and this term basically comes from approximation of kinetic energy operator in nuclear degree of freedom. Further study should be carried out in the near future.

### Concluding remarks

In three out of the four model systems given above, we have shown that the results simulated from the modified semiclassical eq. (6) follow exact electronic coherence as well as amplitude of the overall nonadiabatic transition probabilities, and both the semiclassical Ehrenfest and Tully’s fewest switches methods agree with each other and even for small oscillation in Model 3. On the other hand, the results simulated from the original semiclassical eq. (1) cannot follow exact electronic coherence, and besides the semiclassical Ehrenfest (Tully’s fewest switches) method shows slight greater (smaller) amplitude of the overall nonadiabatic transition probabilities than exact quantum results. This can be seen clearly from Model 3 in the region of monotonically increasing of the overall nonadiabatic transition probabilities against energy. More tests have been performed with changes of potential parameters, and conclusion is the same for the modified coupled Schrödinger equations. The present modified eq. (6) only modifies diagonal element by 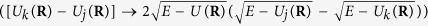 in eq. (1) while preserving original simplicity of Tully’s fewest switches and semiclassical Ehrenfest algorithms. For instance, detailed balance behavior in the present Tully’s fewest switches should follow Tully’s fewest switches and global flux surface hopping^41^, while the present semiclassical Ehrenfest should follow detailed balance of symmetrical quasi-classical treatment of the Meyer-Miller (MM) model^42^. In the multi-state nonadiabatic molecular dynamic simulation, the global flux surface hopping^43^ shows promising accuracy in comparing Tully’s state-to-state surface hopping. This can be immediately applied to the present coupled electronic Schrödinger eq. (6) to perform multi-state nonadiabatic molecular dynamic simulation. However, it should be noticed that when two adiabatic potential energy surfaces significantly degenerate at crossing zone with Renner-Teller coupling, the present modified method still fails. Otherwise, we conclude that in the present theory both semiclassical Ehrenfest and Tully’s fewest switches algorithms are shown to work equally well and the both follow electronic coherence/decoherence the same as exact quantum results. The present theory can be immediately applied to nonadiabatic molecular dynamic simulation for photochemical and photophysical processes involving with electronic excited states.

## Additional Information

**How to cite this article**: Zhu, C. Restoring electronic coherence/decoherence for a trajectory-based nonadiabatic molecular dynamics. *Sci. Rep.* **6**, 24198; doi: 10.1038/srep24198 (2016).

## Supplementary Material

Supplementary Information

## References

[b1] Tully J. C. Molecular dynamics with electronic transitions. J. Chem. Phys. 93, 1601–1701 (1990).

[b2] Delos J. B., Thorson W. R. & Knudson S. K. Semiclassical Theory of Inelastic Collisions. I. Classical Picture and Semiclassical Formulation. Phys. Rev. A 6, 709–719 (1972).

[b3] Iftimie R., Thomas J. W. & Tuckerman M. E. On-the-fly localization of electronic orbitals in Car–Parrinello molecular dynamics. J. Chem. Phys. 120, 2169–2181 (2004). 15268355 10.1063/1.1636697

[b4] Alexandrova A. N., Tully J. C. & Granucci G. Photochemistry of DNA Fragments via Semiclassical Nonadiabatic Dynamics. J. Phys. Chem. B 114, 12116–12128 (2010). 20795696 10.1021/jp103322c

[b5] Yonehara T., Hanasaki K. & Takatsuka K. Fundamental Approaches to Nonadiabaticity: Toward a Chemical Theory beyond the Born Oppenheimer Paradigm. Chem. Rev. 112, 499–542 (2012). 22077497 10.1021/cr200096s

[b6] Clark J., Nelson T., Tretiak S., Cirmi G. & Lanzani G. Femtosecond torsional relaxation. Nat. Phys. 8, 225–231 (2012).

[b7] Heggen B., Lan Z. & Thiel W. Nonadiabatic decay dynamics of 9H-guanine in aqueous solution. Phys. Chem. Chem. Phys. 14, 8137–8146 (2012). 22569748 10.1039/c2cp40300e

[b8] Saita K., Nix M. G. D. & Shalashilin D. V. Simulation of ultrafast photodynamics of pyrrole with a multiconfigurational Ehrenfest method. Phys. Chem. Chem. Phys. 15, 16227–16235 (2013). 23995976 10.1039/c3cp51199e

[b9] Guo X., Zhao Y. & Cao Z. A QM/MM MD insight into photodynamics of hypoxanthine: distinct nonadiabatic decay behaviors between keto-N7H and keto-N9H tautomers in aqueous solution. Phys. Chem. Chem. Phys. 16, 15381–15388 (2014). 24945346 10.1039/c4cp01928h

[b10] Nelson T., Fernandez-Alberti S., Roitberg A. E. & Tretiak S. Nonadiabatic Excited-State Molecular Dynamics: Modeling Photophysics in Organic Conjugated Materials. Acc. Chem. Res. 47, 1155–1164 (2014). 24673100 10.1021/ar400263p

[b11] Xu X., Zheng J., Yang K. R. & Truhlar D. G. Photodissociation Dynamics of Phenol: Multistate Trajectory Simulations including Tunneling. J. Am. Chem. Soc. 136, 16378–16386 (2014). 25348802 10.1021/ja509016a

[b12] Cui G. & Thiel W. Generalized trajectory surface-hopping method for internal conversion and intersystem crossing. J. Chem. Phys. 141, 124101 (2014). 25273406 10.1063/1.4894849

[b13] Yang K. R., Xu X., Zheng J. & Truhlar D. G. Full-dimensional potentials and state couplings and multidimensional tunneling calculations for the photodissociation of phenol. Chem. Sci. 5, 4661–4680 (2014).

[b14] Zimmermann T. & Vaníek J. Efficient on-the-fly ab initio semiclassical method for computing time-resolved nonadiabatic electronic spectra with surface hopping or Ehrenfest dynamics. J. Chem. Phys. 141, 134102 (2014). 25296779 10.1063/1.4896735

[b15] Xia S. H., Xie B. B., Fang Q., Cui G. & Thiel W. Excited-state intramolecular proton transfer to carbon atoms: nonadiabatic surface-hopping dynamics simulations. Phys. Chem. Chem. Phys. 17, 9687–9697 (2015). 25711992 10.1039/c5cp00101c

[b16] Tavernelli I. Nonadiabatic Molecular Dynamics Simulations: Synergies between Theory and Experiments. Acc. Chem. Res. 48, 792–800 (2015). 25647401 10.1021/ar500357y

[b17] Goyal P., Schwerdtfeger C. A., Soudackov A. V. & Hammes-Schiffer S. Nonadiabatic Dynamics of Photoinduced Proton-Coupled Electron Transfer in a Solvated Phenol–Amine Complex. J. Phys. Chem. B 119, 2758–2768 (2015). 25545667 10.1021/jp5126969

[b18] Kab G. Statistical Mechanics of Mean Field Ehrenfest Quantum/Classical Molecular Dynamics: The Damped Harmonic Oscillator. J. Phys. Chem. A 108, 8866–8877 (2004).

[b19] Zhu C., Jasper A. W. & Truhlar D. G. Non-Born-Oppenheimer Liouville-von Neumann Dynamics. Evolution of a Subsystem Controlled by Linear and Population-Driven Decay of Mixing with Decoherent and Coherent Switching. J. Chem. Theory Comput. 1, 527–540 (2005). 26641672 10.1021/ct050021p

[b20] Larsen R. E., Bedard-Hearn M. J. & Schwartz B. J. Exploring the Role of Decoherence in Condensed-Phase Nonadiabatic Dynamics: A Comparison of Different Mixed Quantum/Classical Simulation Algorithms for the Excited Hydrated Electron. J. Phys. Chem. B 110, 20055–20066 (2006). 17020394 10.1021/jp0629745

[b21] Cheng S. C., Zhu C., Liang K. K., Lin S. H. & Truhlar D. G. Algorithmic decoherence time for decay-of-mixing non–Born–Oppenheimer dynamics. J. Chem. Phys. 129, 024112 (2008). 18624521 10.1063/1.2948395

[b22] Subotnik J. E. Fewest-Switches Surface Hopping and Decoherence in Multiple Dimensions. J. Phys. Chem. A 115, 12083–12096 (2011). 21995423 10.1021/jp206557h

[b23] Subotnik J. E. & Shenvi N. A new approach to decoherence and momentum rescaling in the surface hopping algorithm. J. Chem. Phys. 134, 024105 (2011). 21241078 10.1063/1.3506779

[b24] Landry B. R. & Subotnik J. E. How to recover Marcus theory with fewest switches surface hopping: Add just a touch of decoherence. J. Chem. Phys. 137, 22A513 (2012). 10.1063/1.4733675 23249050

[b25] Shenvi N. & Yang W. Achieving partial decoherence in surface hopping through phase correction. J. Chem. Phys. 137, 22A528 (2012). 10.1063/1.4746407 23249065

[b26] Jaeger H. M., Fischer S. & Prezhdo O. V. Decoherence-induced surface hopping. J. Chem. Phys. 137, 22A545 (2012). 10.1063/1.4757100 23249082

[b27] Cotton S. J. & Miller W. H. Symmetrical windowing for quantum states in quasi-classical trajectory simulations: Application to electronically non-adiabatic processes. J. Chem. Phys. 139, 234112 (2013). 24359357 10.1063/1.4845235

[b28] Shenvi N., Subotnik J. E. & Yang W. Phase-Corrected Surface Hopping: Correcting the Phase Evolution of the Electronic Wavefunction. J. Chem. Phys. 135, 024101(2011). 21766919 10.1063/1.3603447

[b29] Nelson T., Fernandez-Alberti S., Roitberg A. E. & Tretiak S. Nonadiabatic excited-state molecular dynamics: Treatment of electronic decoherence. J. Chem. Phys. 138, 224111 (2013). 23781787 10.1063/1.4809568

[b30] Akimov A. V., Long R. & Prezhdo O. V. Coherence penalty functional: A simple method for adding decoherence in Ehrenfest dynamics. J. Chem. Phys. 140, 194107 (2014). 24852530 10.1063/1.4875702

[b31] Cotton S. J., Igumenshchev K. & Miller W. H. Symmetrical windowing for quantum states in quasi-classical trajectory simulations: Application to electron transfer. J. Chem. Phys. 141, 084104 (2014). 25173002 10.1063/1.4893345

[b32] Falk M. J., Landry B. R. & Subotnik J. E. Can Surface Hopping sans Decoherence Recover Marcus Theory? Understanding the Role of Friction in a Surface Hopping View of Electron Transfer. J. Phys. Chem. B 118, 8108–8117 (2014). 24745794 10.1021/jp5011346

[b33] Miller S. C. & Good R. H. A WKB-Type Approximation to the Schrödinger Equation. Phys. Rev. 91, 174–179 (1953).

[b34] Rosen N. & Zener C., Double Stern-Gerlach Experiment and Related Collision Phenomena. Phys. Rev. 40, 502–507 (1932).

[b35] Landau L. D. On the Theory of Transfer of Energy at Collisions II. Phys. Z. Sowjetunion 2, 46–51 (1932).

[b36] Zener C. Non-adiabatic Crossing of Energy Levels. Proc. R. Soc. London A 137, 696–702 (1932).

[b37] Stueckelberg E. C. G. Semiclassical Approximation, Analytical continuation of the WKB Solution Across Stokes Lines. Helv. Phys. Acta 5, 369–422 (1932).

[b38] Renner R. On the Theory of the Interaction between Electronic and Nuclear Motion of Three-atomic Bar-shaped Molecules. Z. Phys. 92, 172–193 (1934).

[b39] Child M. S. Molecular Collision Theory Academic Press, London (1974).

[b40] Desouter-Lecomte M. . Nonadiabatic Unimolecular Reactions of Polyatomic Molecules. J. Phys. Chem. 89, 214–222 (1985).

[b41] Sifain A. E., Wang L. & Prezhdo O. V. Mixed Quantum-Classical Equilibrium in Global Flux Surface Hopping. J. Chem. Phys. 142, 224102 (2015). 26071696 10.1063/1.4922162

[b42] Miller W. H. & Cotton S. J. Communication: Note on Detailed Balance in Symmetrical quasi-classical Models for Electronically Non-adiabatic Dynamics. J. Chem. Phys. 141, 084104 (2015). 10.1063/1.4916945 25854221

[b43] Wang L., Trivedi D. & Prezhdo O. V. Global Flux Surface Hopping Approach for Mixed Quantum-Classical Dynamics. J. Chem. Theory Comput. 10, 3598–3605 (2014). 26588504 10.1021/ct5003835

