# Supplementary material for: Restoring electronic coherence/decoherence for a trajectory-based nonadiabatic molecular dynamics
Source: Sci Rep. 2016 Apr 11;6:24198. doi: 10.1038/srep24198 (PMC4827081; doi:10.1038/srep24198)
Supplement: Supplementary Information [file srep24198-s1.pdf]

Supplementary Information

Restoring electronic coherence/decoherence for a trajectory-based nonadiabatic molecular dynamics

Chaoyuan Zhu

Institute of Molecular Science, Department of Applied Chemistry, and Center for Interdisciplinary Molecular Science, National Chiao-Tung University, Hsinchu 300, Taiwan

Email:cyzhu@mail.nctu.edu.tw

Chapter page

Note 1. Comparing with symmetrical windowing quasi-classical (SQC) approach 2

Note 2. Statistical averaged populations based on trajectories and wave functions 3

Note 3. Comparing with the Gaussian wavepackets phase correlation method 6

Supplementary Note 1. Comparing with symmetrical windowing quasi-classical (SQC) approach

For given a finite set electronic degrees of freedom (F, say), Meyer-Miller classical electronic Hamiltonian is given by [27]

$$H(\mathbf{P},\mathbf{R};\mathbf{p},\mathbf{x})=\frac{\mathbf{P}^2}{2\mu}+\sum_{k=1}^{F}\left(\frac{1}{2}p_k^2+\frac{1}{2}x_k^2-\gamma\right)H_{kk}(\mathbf{R})+\sum_{k'<k=1}^{F}\left(p_k p_{k'}+x_k x_{k'}\right)H_{kk'}(\mathbf{R}), \quad \text{(S1)}$$

where (**P, R**) are the nuclear momenta and coordinates, $\{p_k, x_k\}$ are the Cartesian electronic variables and $H_{k'k}(\boldsymbol{R})$ represents the $F \times F$ diabatic electronic real-symmetric matrix. Test calculation for Dual avoided-crossings (Model 2) with windowing parameter $\gamma = 0.366$ shows very good agreement with exact quantum results for original Tully's potential parameters (A = 0.1, B = 0.28, E_0 = 0.05, C = 0.015, and D = 0.06 in Eq. (8)) [27]. We have recomputed this case with windowing parameter $\gamma = 0.4$ (there is no difference to $\gamma = 0.366$) as shown in Fig. S1. In the case of original Tully's potential parameters, both SQC and the present Ehrenfest methods agree well with exact quantum results as shown in Fig. 1Sa. However, if we modify the two of five parameters as E_0 = 0.03 and C = 0.01 in Eq. (8), Fig. 1Sb shows the present Ehrenfest method works slightly better than SQC method in comparing with exact quantum calculations.

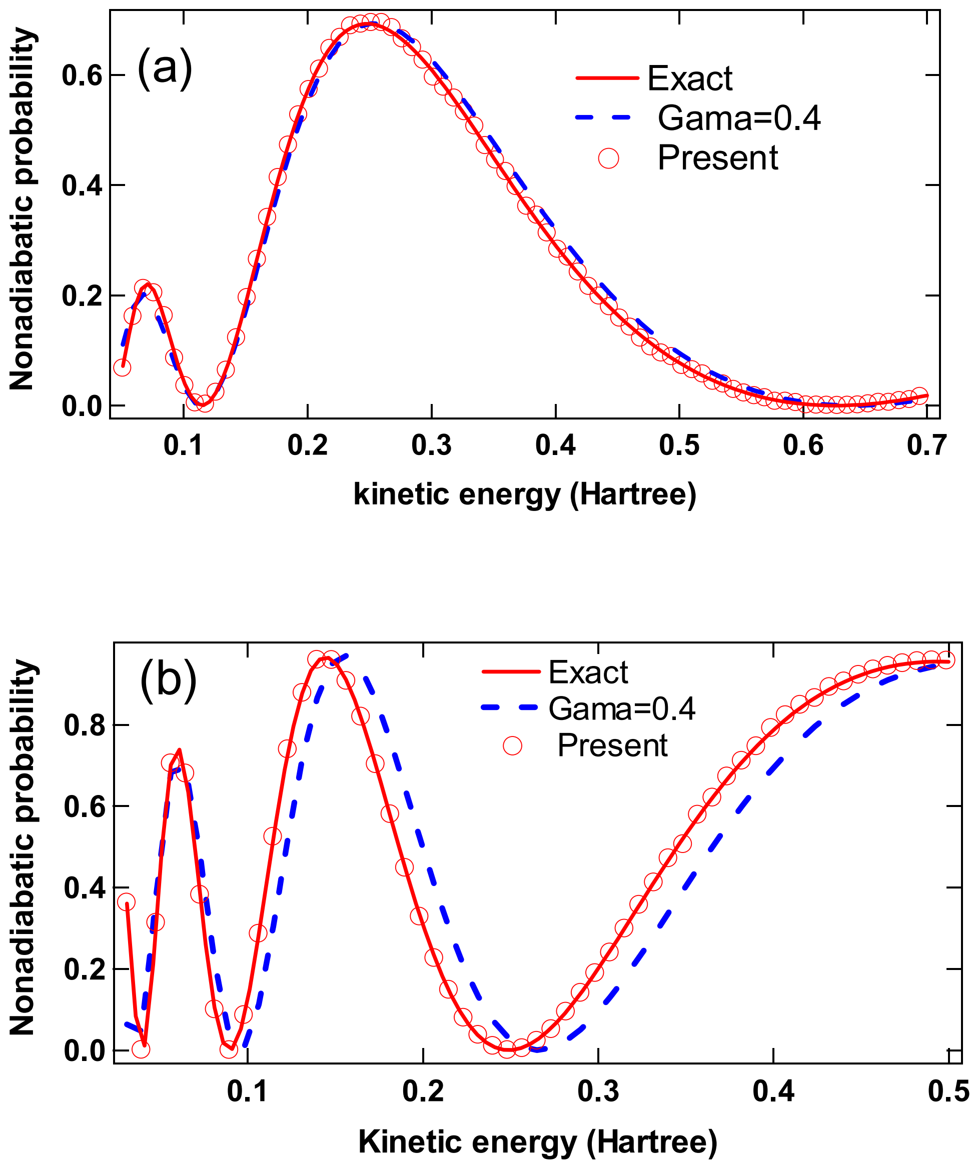

Figure S1. Overall nonadiabatic transition probability (transmission from 1→2) for Model 2. Solid lines represent exact quantum results, dashed line represent SQC calculation and open circles represent the present Ehrenfest calculation. The potential parameters in Eq. (8) are (a) original Tully (A = 0.1, B = 0.28, E_0 = 0.05, C = 0.015, and D = 0.06) and (b) modified (A = 0.1, B = 0.28, E_0 = 0.03, C = 0.01, and D = 0.06).

Supplementary Note 2. Statistical averaged populations based on trajectories and wave functions

The overall nonadiabatic transition probability at given kinetic energy *E* can be

computed based on statistical ensemble of trajectories as follows (let us assume initial state is from electronic state 1 at t = - ∞)

$$P_{1\to 2}(E) = \frac{N_2}{N} \tag{S2}$$

where N_2 represent number of trajectories ending on electronic excited state 2 (at t = + ∞) and N is total number of trajectories in the sampling. For each sampling trajectory, we propagate coupled electronic wave functions (electronic density in Eq. (1) or Eq. (6)) and the other way to compute overall nonadiabatic transition probability can be based on density,

$$P_{1\to 2}(E) = \frac{1}{N}\sum_{i=1}^{N_2} \rho_{22}(i, t = +\infty) \tag{S3}$$

in which $\rho_{22}(i, t = +\infty)$ represents electronic density for the i-th trajectory ending on the state 2.

Before we discuss overall nonadiabatic transition probability simulated from the two statistical averages, we take an example of Model 2. Fig. 1Sb (where E_0 = 0.03 in Eq. (8)) shows more oscillatory structure than Fig. 1Sa (where E_0 = 0.05 in Eq. (8)). Thus, we check even more rapid oscillation case with E_0 = 0.015. Figure 2S shows that both Tully's fewest-switches surface hopping (FSSH) and semiclassical Ehrenfest (SE) work excellently well with the present modified coupled electronic equations.

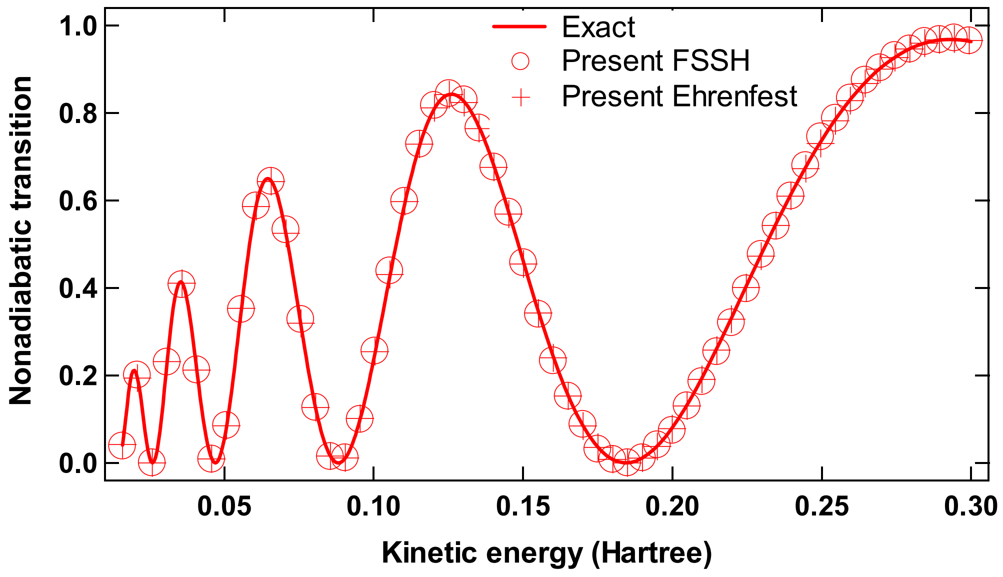

Figure S2. **Overall nonadiabatic transition probability calculated from** Eq. (6) for Model 2 (A = 0.1, B = 0.28, E_0 = 0.015, C = 0.01, and D = 0.06).

Figure S3 shows overall nonadiabatic transition calculated from the present modified coupled electronic Equation (6), and statistical averages from both trajectory-based and wavfunction-based methods all agree with semicalssical Ehrenfest calculation. This confirms that the present modified coupled electronic equations present correct coherent nonadiabatic transition, and thus simulations from FSSH and SE agree well. On the other hand, the coupled electronic equations from Eq. (1) do not present correct coherent nonadiabatic transition, so that simulations from FSSH and SE do not agree as shown in Fig. S4.

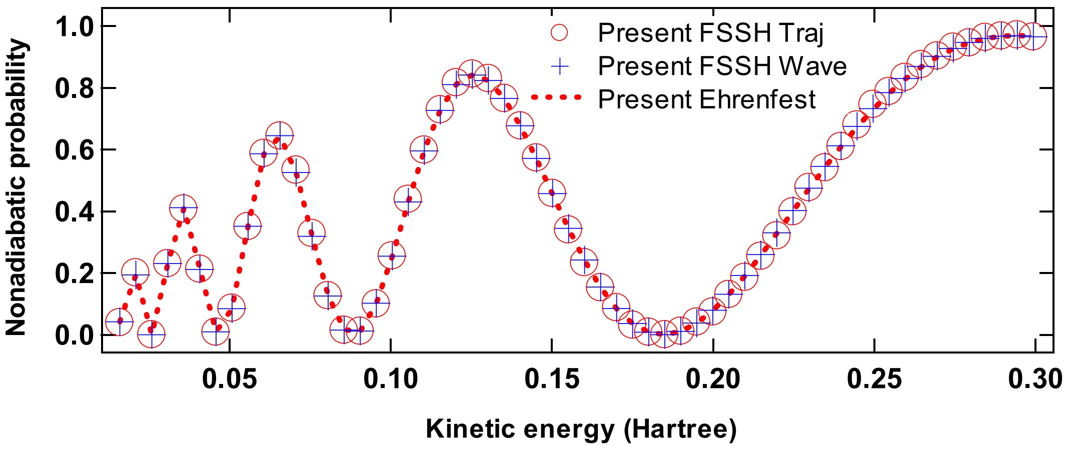

Figure S3. Overall nonadiabatic transition probability calculated from Eq. (6) for Model 2 (A = 0.1, B = 0.28, E_0 = 0.015, C = 0.01, and D = 0.06). (a) Open circles are from Eq. (S2) and (b) cross are from Eq. (S3) based on FSSH method. (c) Dashed lines are based on Ehrenfest method.

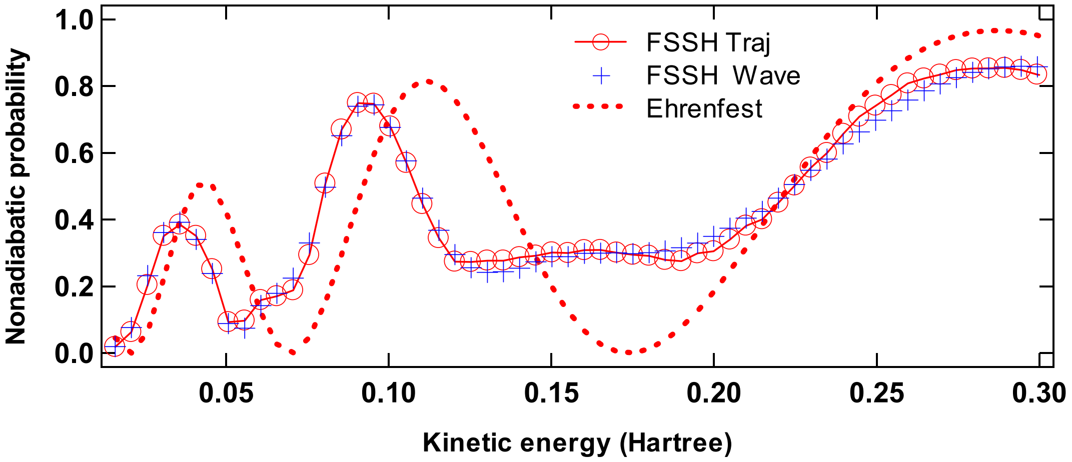

Figure S4. Overall nonadiabatic transition probability calculated from Eq. (1) for Model 2 (A = 0.1, B = 0.28, E_0 = 0.015, C = 0.01, and D = 0.06). (a) Open circles are from Eq. (S2) and (b) cross are from Eq. (S3) based on FSSH method. (c) Dashed lines are based on Ehrenfest method.

Supplementary Note 3. Comparing with the Gaussian wavepackets phase correlation methods

Within Tully's fewest-switches surface hopping (FSSH) framework, Shenvi, Subotnik and Yang [28] consider Gaussian wavepackets as initial condition for coupled electronic Equation (1), then they derive phase correlation between two electronic states

for single particle as

$$\frac{g_1(\mathbf{x}_1)}{g_2(\mathbf{x}_1)} = \exp\left\{\frac{i}{m}\mathbf{p}_1 \cdot (\mathbf{p}_1 - \mathbf{p}_2)\right\}. \tag{S4}$$

For effectively achieving this phase correlation, they wrote it into Hamiltonian in adiabatic representation for coupled electronic wave equations as

$$\begin{pmatrix} -\frac{\mathbf{p}_1 \cdot \mathbf{p}_1}{m} & -i\frac{\hbar}{m}\mathbf{p}_1 \cdot \mathbf{d}_{12} \\ -i\frac{\hbar}{m}\mathbf{p}_1 \cdot \mathbf{d}_{21} & -\frac{\mathbf{p}_1 \cdot \mathbf{p}_2}{m} \end{pmatrix} \leftarrow \text{state 1} \leftrightarrow \text{state 2} \rightarrow \begin{pmatrix} -\frac{\mathbf{p}_1 \cdot \mathbf{p}_2}{m} & -i\frac{\hbar}{m}\mathbf{p}_2 \cdot \mathbf{d}_{12} \\ -i\frac{\hbar}{m}\mathbf{p}_2 \cdot \mathbf{d}_{21} & -\frac{\mathbf{p}_2 \cdot \mathbf{p}_2}{m} \end{pmatrix}. \tag{S5}$$

In the case of one dimension and two-state, Eq. (S5) is the same as the present modified coupled electronic Equation (6). However, if we understand correctly to extend their equation into case with many particles and many states, it goes

$$\begin{pmatrix} -\sum_{\alpha=1}^{N}\frac{\mathbf{p}_i^{\alpha} \cdot \mathbf{p}_i^{\alpha}}{m_\alpha} & -i\hbar\sum_{\alpha=1}^{N}\frac{\mathbf{p}_i^{\alpha} \cdot \mathbf{d}_{ij}^{\alpha}}{m_\alpha} \\ -i\hbar\sum_{\alpha=1}^{N}\frac{\mathbf{p}_i^{\alpha} \cdot \mathbf{d}_{ij}^{\alpha}}{m_\alpha} & -\sum_{\alpha=1}^{N}\frac{\mathbf{p}_i^{\alpha} \cdot \mathbf{p}_j^{\alpha}}{m_\alpha} \end{pmatrix} \tag{S6}$$

for trajectory moving on *i* electronic state hopping for *j* electronic state, and *N* represents number of atoms in the molecule. If we rewrite Eq. (S6) in density representation, diagonal element of Hamiltonian goes to $-\sum_{\alpha=1}^{N}\frac{\mathbf{p}_i^{\alpha} \cdot (\mathbf{p}_i^{\alpha} - \mathbf{p}_j^{\alpha})}{m_\alpha}$, and this is totally different from the present $2\sqrt{E-U}\left[\sqrt{E-U_j} - \sqrt{E-U_i}\right]$ in Eq. (6). The former expression does not go to $(U_i - U_j)$ in Eq. (1) at high kinetic energy limit, while the present form does go to this limit. From Born-Oppenheim approximation with time dependent Schrödinger equation, Tully derives the multi-state coupled electronic Equation (1) and this equation should be correct when kinetic energy is very high. That we consider any modification of

Equation (1) should preserve this limit. From computational point of view, Eq. (S6) needs to calculate momentum vector on the *j*-state while trajectory moving on the *i*-state.

Shenvi, Subotnik and Yang [28] made test calculation for one-particle 2D model system like Dual avoided-crossings as follow:

$$\begin{aligned} &V_{11}(x,y) = 0, \\ &V_{22}(x,y) = -A\exp\left(-B\left(x^2+y^2+xy\right)\right)+E_0, \\ &V_{12}(x,y) = V_{21}(x,y) = C\exp\left(-D\left(x^2+y^2-xy\right)\right), \end{aligned} \tag{S7}$$

with potential parameters (A = 0.15, B = 0.14, E_0 = 0.05, C = 0.015, and D = 0.06). Their calculation shows that overall nonadiabatic transition probability does follow exact quantum oscillation correctly as shown in Fig. S5. Our calculation with the present coupled electronic Equation (6) follows exact quantum oscillation correctly as well.

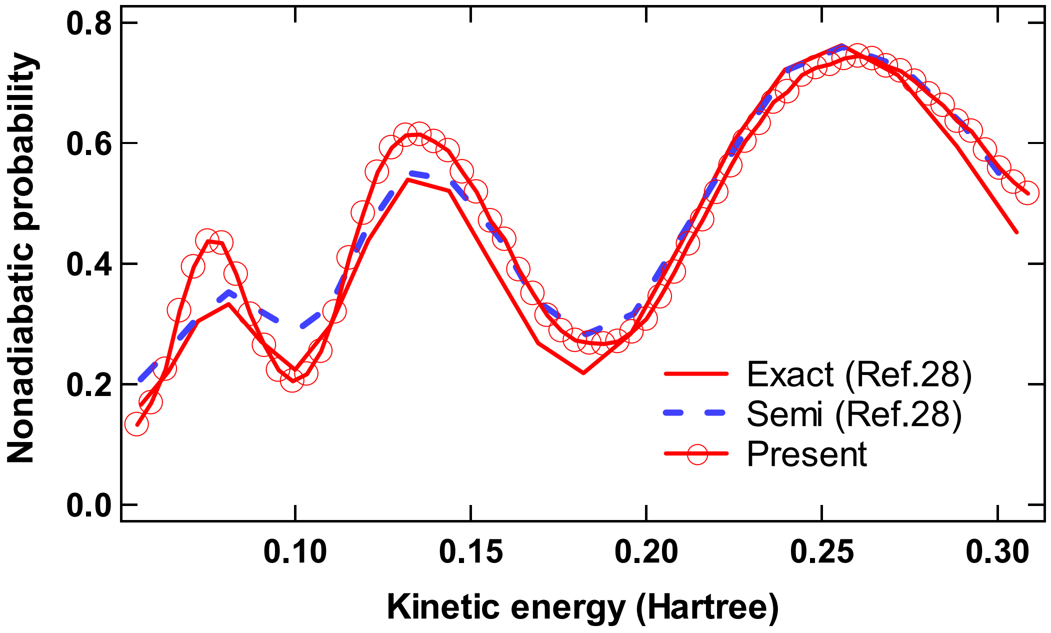

Figure S5. Overall nonadiabatic transition probability (transmission from 1→2) for model system of Eq. (S7). Solid lines represent exact quantum results (Ref. 28) and dashed lines represent FSSH results from Eq. (S5). Open circles represent FSSH results from Eq. (6).
